# Supplementary material for: Role and mechanism of NCAPD3 in promoting malignant behaviors in gastric cancer
Source: Front Pharmacol. 2024 Apr 22;15:1341039. doi: 10.3389/fphar.2024.1341039 (PMC11070777; doi:10.3389/fphar.2024.1341039)
Supplement: Supplementary file 11 [file DataSheet2.ZIP › GSEA/Canonical pathways/my_analysis.Gsea.1599462267220/REACTOME_SLC_MEDIATED_TRANSMEMBRANE_TRANSPORT.html]

Details for gene set REACTOME\_SLC\_MEDIATED\_TRANSMEMBRANE\_TRANSPORT[GSEA]

|  || Dataset | filtered\_dataset.sample\_info.cls#WT\_versus\_NCAPD3\_MUT |
| Phenotype | sample\_info.cls#WT\_versus\_NCAPD3\_MUT |
| Upregulated in class | NCAPD3\_MUT |
| GeneSet | REACTOME\_SLC\_MEDIATED\_TRANSMEMBRANE\_TRANSPORT |
| Enrichment Score (ES) | -0.39461395 |
| Normalized Enrichment Score (NES) | -1.9521762 |
| Nominal p-value | 0.0026041667 |
| FDR q-value | 0.027731117 |
| FWER p-Value | 0.156 |
Table: GSEA Results Summary

  

Fig 1: Enrichment plot: REACTOME\_SLC\_MEDIATED\_TRANSMEMBRANE\_TRANSPORT      
 Profile of the Running ES Score & Positions of GeneSet Members on the Rank Ordered List

  

| SYMBOL | TITLE | RANK IN GENE LIST | RANK METRIC SCORE | RUNNING ES | CORE ENRICHMENT || 1 | 1497 | CTNS | 58 | 0.883 | 0.0203 | No |
| 2 | 55343 | SLC35C1 | 92 | 0.804 | 0.0531 | No |
| 3 | 55676 | SLC30A6 | 353 | 0.565 | -0.0946 | No |
| 4 | 23315 | SLC9A8 | 398 | 0.537 | -0.0885 | No |
| 5 | 801 | CALM1 | 424 | 0.518 | -0.0701 | No |
| 6 | 6507 | SLC1A3 | 464 | 0.496 | -0.0634 | No |
| 7 | 81031 | SLC2A10 | 539 | 0.453 | -0.0849 | No |
| 8 | 51000 | SLC35B3 | 740 | 0.355 | -0.2040 | No |
| 9 | 57181 | SLC39A10 | 811 | 0.313 | -0.2325 | No |
| 10 | 6558 | SLC12A2 | 839 | 0.276 | -0.2326 | No |
| 11 | 144195 | SLC2A14 | 872 | -0.283 | -0.2357 | No |
| 12 | 6515 | SLC2A3 | 873 | -0.283 | -0.2159 | No |
| 13 | 6520 | SLC3A2 | 878 | -0.290 | -0.1984 | No |
| 14 | 8140 | SLC7A5 | 890 | -0.305 | -0.1848 | No |
| 15 | 54407 | SLC38A2 | 1182 | -0.553 | -0.3557 | Yes |
| 16 | 23657 | SLC7A11 | 1205 | -0.581 | -0.3307 | Yes |
| 17 | 861 | RUNX1 | 1293 | -0.687 | -0.3451 | Yes |
| 18 | 8501 | SLC43A1 | 1296 | -0.690 | -0.2980 | Yes |
| 19 | 57835 | SLC4A5 | 1319 | -0.736 | -0.2620 | Yes |
| 20 | 6509 | SLC1A4 | 1330 | -0.752 | -0.2164 | Yes |
| 21 | 7779 | SLC30A1 | 1332 | -0.755 | -0.1639 | Yes |
| 22 | 9990 | SLC12A6 | 1379 | -0.922 | -0.1322 | Yes |
| 23 | 6541 | SLC7A1 | 1388 | -0.981 | -0.0689 | Yes |
| 24 | 6519 | SLC3A1 | 1404 | -1.195 | 0.0043 | Yes |
Table: GSEA details [plain text format]

  

Fig 2: REACTOME\_SLC\_MEDIATED\_TRANSMEMBRANE\_TRANSPORT      
 Blue-Pink O' Gram in the Space of the Analyzed GeneSet

  

Fig 3: REACTOME\_SLC\_MEDIATED\_TRANSMEMBRANE\_TRANSPORT: Random ES distribution      
 Gene set null distribution of ES for **REACTOME\_SLC\_MEDIATED\_TRANSMEMBRANE\_TRANSPORT**
